# Increased 1-aminocyclopropane-1-carboxylate deaminase activity enhances *Agrobacterium tumefaciens*-mediated gene delivery into plant cells

**DOI:** 10.1002/mbo3.123

**Published:** 2013-09-02

**Authors:** Tatsuhiko Someya, Satoko Nonaka, Kouji Nakamura, Hiroshi Ezura

**Affiliations:** Graduate School of Life and Environmental Sciences, University of Tsukuba1-1-1 Tennodai, Tsukuba-shi, Ibaraki, 305-8572, Japan

**Keywords:** ACC deaminase, *Agrobacterium*, promoter, T-DNA delivery

## Abstract

*Agrobacterium*-mediated transformation is a useful tool for the genetic modification in plants, although its efficiency is low for several plant species. *Agrobacterium*-mediated transformation has three major steps in laboratory-controlled experiments: the delivery of T-DNA into plant cells, the selection of transformed plant cells, and the regeneration of whole plants from the selected cells. Each of these steps must be optimized to improve the efficiency of *Agrobacterium*-mediated plant transformation. It has been reported that increasing the number of cells transformed by T-DNA delivery can improve the frequency of stable transformation. Previously, we demonstrated that a reduction in ethylene production by plant cells during cocultivation with *A. tumefaciens*-expressing 1-aminocyclopropane-1-carboxylic acid (ACC) deaminase resulted in increased T-DNA delivery into the plant cells. In this study, to further improve T-DNA delivery by *A. tumefaciens*, we modified the expression cassette of the ACC deaminase gene using *vir* gene promoter sequences. The ACC deaminase gene driven by the *virD1* promoter was expressed at a higher level, resulting in a higher ACC deaminase activity in this *A. tumefaciens* strain than in the strain with the *lac* promoter used in a previous study. The newly developed *A. tumefaciens* strain improves the delivery of T-DNA into *Solanum lycopersicum* (tomato) and *Erianthus ravennae* plants and thus may be a powerful tool for the *Agrobacterium*-mediated genetic engineering of plants.

## Introduction

*Agrobacterium tumefaciens* is a phytopathogenic, soilborne, Gram-negative bacterium that is used to introduce transgenes into plant genomes. *Agrobacterium*-mediated transformation is an important technique in plant science research. The process of *Agrobacterium*-mediated transformation is divided into three steps: (1) the delivery of T-DNA into plant cells via *A*. *tumefaciens*, (2) the selection of transformed cells by antibiotics and the resistance marker genes, and (3) the regeneration of whole plants from the selected cells. Although this *Agrobacterium*-mediated transformation is well established for model plants, it remains ineffective in many plant species of practical importance. It is necessary to improve the efficiency of each step to generate sufficient numbers of transformed plants for evaluation.

*Agrobacterium*-mediated T-DNA delivery into plant cells occurs via the integration of T-DNA from a tumor-inducing (Ti) plasmid in *A. tumefaciens* into the host plant cells (reviewed in Tzfira et al. 2004). The virulence genes that are essential for T-DNA delivery are regulated by the two-component VirA/VirG system and a plant-derived phenolic compound such as acetosyringone (Stachel et al. 1985, reviewed in Pitzschke and Hirt 2010). It has been reported that certain plant hormones, such as salicylic acid, ethylene, cytokinin, auxin, and abscisic acid, induce defense responses against *Agrobacterium*-mediated plant transformation (Davis et al. 1992; Yuan et al. 2007; Lee et al. 2009; Hwang et al. 2010; Rico et al. 2010). For instance, salicylic acid interferes with the ability of *A. tumefaciens* to infect plants by suppressing the transcription of the *vir* genes, the *repABC* operon, and genes associated with quorum sensing (Anand et al. 2008). In addition, ethylene represses *vir* gene expression during transformation (Nonaka et al. 2008a). Consequently, ethylene functions as a repressor that inhibits *Agrobacterium*-mediated gene transfer (Ezura et al. 2000; Han et al. 2005).

We previously constructed an *A. tumefaciens* harboring a plasmid-encoding 1-aminocyclopropane-1 carboxylate, enzyme devoted to the degradation of the immediate precursor of ethylene (Nonaka et al. 2008b). The ACC deaminase-expressing *A. tumefaciens* strain suppressed ethylene synthesis and enhanced the gene transfer efficiency into melon cotyledon cells (Nonaka et al. 2008b). It has also been reported that the ACC deaminase-expressing *A. tumefaciens* strain allows for the efficient genetic transformation of the “Egusi” melon (Ntui et al. 2010). Furthermore, a similar *A. tumefaciens* strain with ACC deaminase activity was constructed and was also shown to be effective for the transformation of canola cultivars (Hao et al. 2010). Thus, the repression of ethylene synthesis in plant cells during T-DNA delivery is an effective method to efficiently generate transgenic plants using *A. tumefaciens*.

In this study, to improve the ACC deaminase-expressing *A. tumefaciens* strain, we modified the ACC deaminase expression cassette. Previously, the ACC deaminase gene in the pBBRacdS plasmid was expressed under the control of the *lac* promoter of *Escherichia coli* (Nonaka et al. 2008b), which exhibits transcriptional activity in acidic media (Chen and Winans 1991). Klüsener et al. (2010) showed that the expression levels of several *A. tumefaciens* virulence genes were the highest in acidic media (AB medium at pH 5.5) containing 100-μmol/L acetosyringone (Klüsener et al. 2010). It is speculated that the promoters of the virulence genes would exhibit higher transcriptional activity than the *lac* promoter in *A. tumefaciens* upon cocultivation with plants. Therefore, to express high levels of ACC deaminase in *A. tumefaciens* during cocultivation with plants, the *lac* promoter sequence should be substituted with virulence gene promoter sequences that permit a high level of gene expression under these particular culture conditions. In this study, we demonstrated that an *A. tumefaciens* strain harboring a plasmid with a virulence gene promoter for the expression of ACC deaminase resulted in an increased efficiency of *Agrobacterium*-mediated transient transformation in *Solanum lycopersicum*, and *Erianthus ravennae* plants relative to that for *A. tumefaciens* harboring the pBBRacdS plasmid with a *lac* promoter.

## Materials and Methods

### Bacterial strains and culture conditions

The bacterial strains used in this study are listed in Table 1. *E. coli* strains were grown at 37°C in LB medium (1% Bacto-Tryptone, 0.5% yeast extract, 0.5% NaCl). *A. tumefaciens* strains were grown at 28°C in LB medium or Murashige and Skoog (MS) medium (Murashige and Skoog 1962). *E. coli* (pQE60) was cultured in LB medium containing 100 mg/L ampicillin and 20 mg/L kanamycin. *A. tumefaciens* (pBBR1MCS5 and the derivative strain pIG121-Hm) was selected with 100 mg/L ampicillin, 50 mg/L gentamicin, and 100 mg/L kanamycin. Gentamicin (50 mg/L), ampicillin (100 mg/L), and spectinomycin (50 mg/L) were used for the selection of *A. tumefaciens* (pBBR1MCS5 and the derivative strain pEKH_2_).

**Table 1:** Plasmids and strains used in this study

	Description	Reference
Strain		
GV2260	Nononcogenic *A. tumefaciens* strain	Deblaere et al. (1985)
MCS	GV2260 containing the pBBR1MCS5 plasmid	Nonaka et al. (2008a,b)
acdS	GV2260 containing the pBBRacdS plasmid	Nonaka et al. ([Bibr b16][Bibr b17])
virB1acdS	GV2260 containing the pvirB1acdS plasmid	This study
virD1acdS	GV2260 containing the pvirD1acdS plasmid	This study
virE1acdS	GV2260 containing the pvirE1acdS plasmid	This study
Plasmid		
pQEAcdS	Overexpression vector for ACC deaminase; Amp^R^	This study
pBBR1MCS-5	Broad host-range shuttle vector; Gen^R^	Kovach et al. (1995)
pBBRacdS	Overexpression vector for ACC deaminase under the control of the *lac* promoter; Gm^R^	Nonaka et al. ([Bibr b16][Bibr b17])
pvirB1acdS	Overexpression vector for ACC deaminase under the control of the *virB1* promoter; Gm^R^	This study
pvirD1acdS	Overexpression vector for ACC deaminase under the control of the *virD1* promoter; Gm^R^	This study
pvirE1acdS	Overexpression vector for ACC deaminase under the control of the *virE1* promoter; Gm^R^	This study
pIG121-Hm	Binary vector plasmid carrying the b*-*glucuronidase gene (*gusA*) between the T-borders; Km^R^	Ohta et al. (1990)
pEKH_2_	Binary vector plasmid carrying the b*-*glucuronidase gene (*gusA*) between the T-borders; Sp^R^	Hoshikawa et al. (2012)

### Construction of plasmids

The primers and plasmids used in this study are listed in Table S1 and Table 1, respectively. The transcriptional fusion plasmids were constructed as follows: to delete the restriction site downstream of the *acdS* gene in the pBBRacdS plasmid, an *acdS* fragment was amplified with the primers acdS-*Nco*I and acdS-*Spe*I. The resulting product was digested with *Nco*I and *Spe*I and then ligated to an *Nco*I*-* and *Xba*I-digested pBBR1MCS-5 vector. The *lacZ-acdS* ORF fragment was then amplified by the primers plac-ATG and acdS_*Eco*RI. The DNA fragments of various *vir* promoter regions, including the *vir* box, were amplified by PCR using the genomic DNA of *A. tumefaciens* as the template. Each of the amplified promoter fragments and the *lacZ-acdS* ORF fragment was ligated by fusion PCR, and these fusion fragments were then digested with *Nco*I and *Sca*I and cloned into the pBBR1MCS-5 vector. The overexpression vector, designated pQE60AcdS, for the C-terminal 6× His AcdS fusion protein (AcdS-His) was constructed by inserting a PCR-amplified *acdS* gene into the *Nco*I-*Bgl*II-digested pQE60 expression vector (Qiagen, Hamburg, Germany). The PCR product was obtained using the pQEacdS-F and pQEacdS-R primers.

### Antibody preparation

The expression of AcdS-His in *E. coli* M15/pREP4 was induced for 5 h in the presence of 1 mmol/L IPTG. The cells were harvested by centrifugation at 5000 *g* at 4°C for 15 min. The wet cells were suspended in 10 mL of lysis buffer (50-mmol/L NaH_2_PO_4_, 300-mmol/L NaCl, pH 8.0), lysed on ice by sonication, and centrifuged at 5000 × *g* at 4°C for 15 min. The supernatant was incubated with His-select™ Nickel Affinity gel (Sigma, St. Louis, MO) at 4°C for 1 h and eluted with elution buffer (50-mmol/L NaH_2_PO_4_, 300-mmol/L NaCl, 250-mmol/L imidazole, pH 8.0). The purity of the AcdS-His protein was analyzed by 15% SDS-PAGE. The eluted sample was dialyzed against 50-mmol/L Tris-HCl buffer (pH 8.0) containing 100-mmol/L NaCl and 50% glycerol. The AcdS-His fusion protein was injected into rabbits to prepare polyclonal antibodies.

### Western blots

The samples were prepared from whole-cell extracts of strains grown in MS medium containing 200-μmol/L acetosyringone. The cells were harvested by centrifugation at 5000 × *g* at 4°C for 15 min. Wet cells were suspended in lysis buffer (100-mmol/L Tris-HCl, 0.1% SDS, 0.1% Triton X-100, pH 8.5), lysed on ice by sonication, and centrifuged at 5000 × *g* at 4°C for 15 min. Equivalent volumes containing 0.03 OD_600_ units of protein were resolved by SDS-PAGE and then electroblotted onto polyvinylidene difluoride membranes. The membranes were blocked with 5% skim milk in TBS-containing 0.2% Tween 80. Primary and secondary antibodies were diluted in TBS-containing 0.2% Tween 80. The primary antibody was a rabbit polyclonal anti-ACC deaminase antibody, and the secondary antibody was an HRP-conjugated donkey anti-rabbit IgG. Bound antibodies were then detected using ImmunoSar LD (Wako, Tokyo, Japan).

### ACC deaminase activity assay

Cells were collected and washed twice with 100-mmol/L Tris-HCl (pH 8.5) and resuspended in 1.5 mL of lysate buffer. The cells were lysed on ice by sonication and centrifuged at 5000 × *g* at 4°C for 15 min. The ACC deaminase activity was measured according to a modified protocol based on that of Honma and Shimomura (1978). The ACC deaminase activity was measured spectrophotometrically at 340 nm. The protein content of the extracts was determined using the Bradford method (Bradford 1976).

### Plant material

Seeds of *S. lycopersicum* cv. Moneymaker were washed with 70% ethanol for 10 sec, sterilized with 5% hypochlorous acid containing 10% Triton X-100 for 45 min, and washed three times with sterilized water. After the third wash, the seeds were kept in water overnight. The sterilized *S. lycopersicum* seeds were sown on MS medium containing 15 g/L sucrose (Wako) and 3% gellan gum (Wako) and then grown for 7 days. *E. ravennae* calli, which were induced from seeds on MS medium-containing 1 g/L casamino acids, 2 mg/L 2,4-D, 0.2 mg/L 6-benzylaminopurine (BAP), 30 g/L maltose H (Wako), and 3% gellan gum, were kindly provided by Prof. Masashiro Mii of Chiba University in Japan. The calli were subcultured for 2 weeks before *A. tumefaciens* inoculation.

### *Agrobacterium*-mediated T-DNA transfer

#### Preparation of *A. tumefaciens*

*Agrobacterium tumefaciens* was cultured on solid LB medium at 28°C for 2 days. A single colony was picked and cultured in 2 mL of LB medium at 28°C and 200 rpm for 2 days until the culture reached the stationary phase. From this culture, 15 μL was harvested and added to 15 mL of LB medium, which was then cultured at 28°C and 200 rpm for 20 h. When the optical density of the culture reached 0.8-1.0, the cells were centrifuged, and the pelleted bacterial cells were resuspended in liquid MS medium containing 30 g/L glucose and 200 μmol/L acetosyringone at pH 5.2. The optical density was adjusted to 0.4-0.5.

#### *Solanum lycopersicum* cv. Moneymaker

Cotyledons from 7-day-old *S. lycopersicum* seedlings were cut into four explants and used to generate two locations for inoculation with *A. tumefaciens*. Thirty explants were subjected to each treatment. The inoculated explants were cultured on cocultivation medium, which contained MS salts, 30 g/L glucose, 200 μmol/L acetosyringone (pH 5.2), and 3% gellan gum, at 25°C for 3 days in the dark. After 3 days of cocultivation, the *S. lycopersicum* explants were assayed histochemically for GUS activity with X-Gluc buffer.

#### Erianthus ravennae

*Erianthus ravennae* calli were inoculated with *A. tumefaciens* and cultured on MS medium containing 1 g/L casamino acids, 30 g/L glucose, 3% gellan gum, 2 mg/L 2,4-D, and 200-μmol/L acetosyringone at pH 5.2 for 3 days. After 3 days of cocultivation, the calli were washed with sterilized water and transferred to MS medium containing 1 g/L casamino acids, 2 mg/L 2,4-D, 0.2 mg/L BAP, 30 g/L glucose, 3% gellan gum, and 12.5 mg/L meropenem trihydrate (Dainippon Sumitomo Pharma) for 1 day to eliminate *A. tumefaciens*. The inoculated calli were assayed using histochemistry and quantitative activity assays.

### Estimation of the T-DNA transfer efficiency

#### *Solanum lycopersicum* cv. Moneymaker

After 3 days of cocultivation, *S. lycopersicum* segments were placed in GUS staining buffer-containing 100-mmol/L phosphate buffer, 10-mmol/L EDTA, 2.5-mmol/L potassium ferricyanide, 2.5-mmol/L potassium ferrocyanide, 0.1% Triton X-100, and 0.5 mg/L X-glucuronide. GUS-stained *S. lycopersicum* cotyledon explants were observed, and images were taken using a stereoscopic microscope system (Leica: MX FLIII, DFC300 FX, Application Suite, Leica, Germany). The GUS-stained area was converted into a numerical value using Image J (National Institutes of Health: http://rsbweb.nih.gov/ij/), and the percentage of GUS-stained area was calculated for each explant. According to these results, the GUS-stained *S. lycopersicum* explants were categorized into six classes (less than 1%, 1% to 3%, 3% to 5%, 5% to 10%, 10% to 20%, and more than 20%). To estimate the T-DNA transformation efficiency, the frequency of each class was calculated.

#### Erianthus ravennae

After cocultivation, the GUS activity of *E. ravennae* calli was assayed histochemically with GUS staining buffer as described above. The GUS-stained calli were observed using a stereoscopic microscope, and the number of GUS-stained spots was counted. After counting, the number of GUS-stained spots per 1 g of calli was calculated. The T-DNA transfer efficiency was estimated based on the relative number of GUS spots.

The T-DNA transfer efficiency was also estimated based on the quantitative GUS activity measured using the fluorometric assay previously described by Nonaka et al. (2008b) with a Perkin-Elmer ARYO MX-FL 1420 Multilabel Counter fluorometer (Perkin-Elmer, Waltham, MA) and 4-methylumbelliferyl beta-d-glucuronide (MUG) as the substrate.

## Results and Discussion

### Effects of different promoters on the expression of ACC deaminase

**Figure 1:**
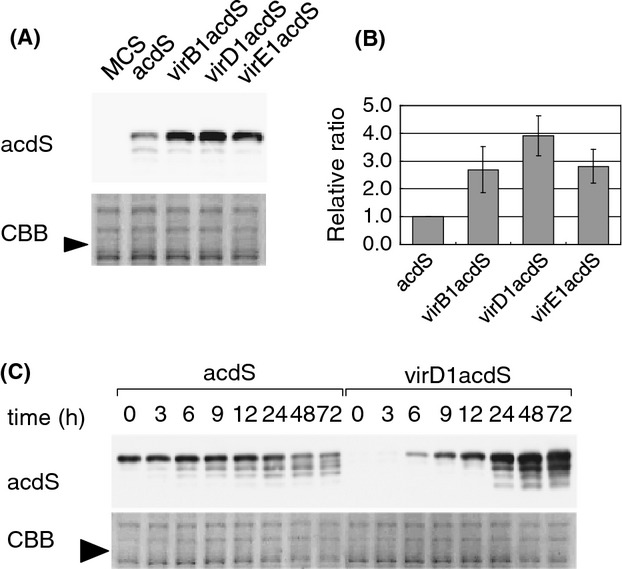
Semiquantitative analysis of ACC deaminase expression in *A. tumefaciens* strains. (A) Western blot analysis of ACC deaminase expression was performed using cell extracts. ACC deaminase was probed with an anti-ACC deaminase antibody. Coomassie Brilliant Blue staining (bottom panel) is shown as an internal control. Arrow head indicates 57kDa. (B) Relative intensities of the immunoreactive signals from the Western blots in (A). The ACC deaminase expression level in the *A. tumefaciens* GV2260 (pBBRacdS) strain was set to 1.0. The error bars show the standard deviation (*n* = 3). (C) Western blot analysis of ACC deaminase after 0–72 h of subculture on MS medium with acetosyringone [left panel: *A. tumefaciens* GV2260 (pBBRacdS); right panel: *A. tumefaciens* GV2260 (pvirD1acdS)]. MCS: *A. tumefaciens* GV2260 (pBBRMCS1-5); acdS: *A. tumefaciens* GV2260 (pBBRacdS); virB1acdS: *A. tumefaciens* GV2260 (pvirB1acdS); virD1acdS: *A. tumefaciens* GV2260 (pvirD1acdS); virE1acdS: *A. tumefaciens* GV2260 (pvirE1acdS). Arrow head means 57kDa.

The ACC deaminase gene in the pBBRacdS plasmid was expressed under the control of the *lac* promoter from *E. coli* (Nonaka et al. 2008b). To increase the expression of ACC deaminase, we constructed transcriptional fusion plasmids. The promoter sequences originated from *A. tumefaciens* and were selected due to their high expression of genes in *A. tumefaciens* during cocultivation with plant cells. Klüsener et al. (2010) reported that the *virB1* operon, *virD1* operon, and *virE1* operon, which *vir* genes were induced in response to acetosyringone, and therefore we attempted to utilize the *vir* gene promoters to increase compared to ACC deaminase expression and the activity in *A. tumefaciens* cells. We constructed three plasmids with these promoters driving ACC deaminase expression. These plasmids included the VirG-binding site upstream of the promoter sequence (Steck et al. 1988). *A. tumefaciens* strains harboring these expression plasmids were cultured in liquid MS medium-containing 200-μmol/L acetosyringone for 14 h, and the expression of ACC deaminase in the whole-cell lysates was measured by Western blotting (Fig. 1A). The expression of ACC deaminase driven by the virulence gene promoters was greater than that from the pBBRacdS plasmid with the *lac* promoter (Fig. 1A,B). The expression of ACC deaminase increased by fourfold in the *A. tumefaciens* GV2260 (pvirD1acdS) strain (Fig. 1A,B). Next, we analyzed the time course (0–72 h) of acetosyringone-induced ACC deaminase accumulation in MS medium. In the *A. tumefaciens* GV2260 (pvirD1acdS) strain, ACC deaminase expression was induced at 6 h by adding acetosyringone, and high expression was still observed at 72 h (Fig. 1C). In contrast, the expression of ACC deaminase decreased in the *A. tumefaciens* GV2260 (pBBRacdS) strain (Fig. 1C). The expressed protein in both strains containing either plasmid was degraded by endogenous proteases.

**Figure 2:**
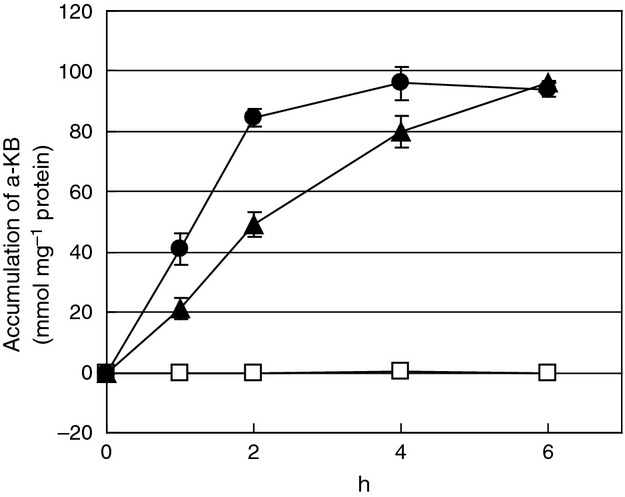
1-aminocyclopropane-1-carboxylic acid deaminase activity for various expression cassettes in *A. tumefaciens*. The accumulation of α-ketobutyrate in cell lysates was determined as described in the Materials and Methods section. The open squares, closed triangles, and closed circles indicate the *A. tumefaciens* GV2260 (pBBR1MCS-5), *A. tumefaciens* GV2260 (pBBRacdS), and *A. tumefaciens* GV2260 (pvirD1acdS) strains, respectively. The values were standardized to the value at 0 h. The error bars show the standard deviation (*n* = 3).

In addition, we determined whether the expressed ACC deaminase was active. ACC deaminase catalyzes the degradation of ACC to α-ketobutyric acid and ammonia. We determined the level of α-ketobutyric acid using the whole-cell lysates from cells that had been cultured in MS medium containing 200-μmol/L acetosyringone. The amounts of α-ketobutyric acid after a 1-h reaction time for *A. tumefaciens* GV2260 (pBBRacdS) and *A. tumefaciens* GV2260 (pvirD1acdS) were 21.3 ± 3.3 and 41.1 ± 5.3 μmol/mg protein, respectively (Fig. 2). This result indicates that the ACC deaminase expressed from the pvirD1acdS plasmid was enzymatically active in the cells, and the *A. tumefaciens* GV2260 (pvirD1acdS) strain showed higher ACC deaminase activity than the *A. tumefaciens* GV2260 (pBBRacdS) strain.

### Efficiency of T-DNA delivery

#### *Solanum lycopersicum* cv. Moneymaker

**Figure 3:**
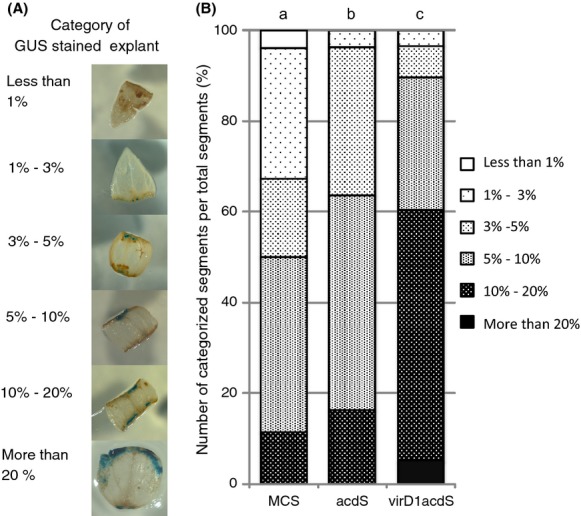
Estimation of the *Agrobacterium*-mediated T-DNA transfer efficiency in *S. lycopersicum* plants. (A) Classification of GUS-stained cotyledon explants. GUS-stained *S. lycopersicum* cotyledons were categorized based on the stained area: less than 1%, 1% to 3%, 3% to 5%, 5% to 10%, 10% to 20%, and more than 20%. (B) The frequency of each GUS staining category in *S. lycopersicum* explants. Bacterial strains resulting in significant differences (Student's *T*-test and Kruskal–Wallis test; *P* < 0.01) are indicated by different letters. MCS: *A. tumefaciens* GV2260 (pBBRMCS1-5, pIG121-Hm); acdS: *A. tumefaciens* GV2260 (pBBRacdS, pIG121-Hm); virD1acdS: *A. tumefaciens* GV2260 (pvirD1acdS, pIG121-Hm).

To evaluate *Agrobacterium*-mediated T-DNA delivery, 60–80 *S. lycopersicum* explants from 7-day-old seedlings were prepared and inoculated with *A. tumefaciens*. Three types of *A. tumefaciens* strains [*A. tumefaciens* GV2260 (pBBR1MCS-5, pIG121-Hm), *A. tumefaciens* GV2260 (pBBRacdS, pIG121-Hm), and *A. tumefaciens* GV2260 (pvirD1acdS, pIG121-Hm)] were used in this experiment. The *uidA* gene was used as an indicator of T-DNA delivery, and the efficiency of T-DNA delivery was estimated by GUS staining. We determined the GUS-stained area in each of the explants with Image J, as described in the Materials and Methods. The degree of staining was categorized into six classes (Fig. 3A), and the frequency of each class was calculated (Fig. 3). This experiment was repeated three times. *A. tumefaciens* GV2260 (pBBRacdS, pIG121-Hm) decreased the frequency of *S. lycopersicum* explants with a low degree of staining (less than 5%) relative to the frequency for inoculation with *A. tumefaciens* GV2260 (pBBRMCS, pIG121-Hm) (Fig. 3B). Compared with *A. tumefaciens* GV2260 (pBBRacdS, pIG121-Hm), *A. tumefaciens* GV2260 (pvirD1acdS, pIG121-Hm) increased the frequency of high staining (more than 10%) and decreased the frequency of low staining (less than 5%) (Fig. 3B). All these results showed the same tendency in three repetitions. Therefore, the increases in ACC deaminase gene expression and activity improved the delivery of T-DNA by *A. tumefaciens*, and we succeeded in developing an *A. tumefaciens* strain capable of effectively transforming *S. lycopersicum* cells.

#### Erianthus ravennae

**Figure 4:**
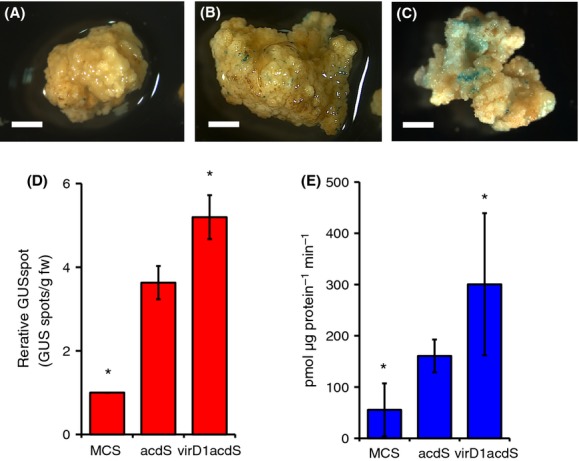
T-DNA delivery efficiency in *E. ravennae*. GUS-stained calli inoculated with *A. tumefaciens* (pBBRMCS1-5, pEKH_2_) (A), *A. tumefaciens* (pBBRacdS, pEKH_2_) (B), and *A. tumefaciens* (pviracdS, pEKH_2_) (C). Blue spots indicate transformed cells. The bar indicates 5 mm. (D) Occurrence of T-DNA transformation in *E. ravennae*. This graph shows the relative number of GUS spots. The number of GUS-stained spots per 1 g of *E. ravennae* calli was counted for each treatment. The bars indicate the standard deviation (*n* = 3). Asterisks indicate values that were significantly different from *A. tumefaciens* (pEKH_2_, pBBRacdS) inoculation according to the Student's T-test (*P* < 0.05). (E) Estimation of the transformation efficiency using the fluorometric GUS assay. GUS activity was measured immediately after cocultivation. The mean GUS activity ± SD was calculated from the results of three experiments. Approximately 2 g of *E. ravennae* calli was used in each experiment. Asterisks indicate statistically significant differences according to Student's *T*-test (*P* < 0.05). MCS: *A. tumefaciens* GV2260 (pBBRMCS1-5, pEKH_2_); acdS: *A. tumefaciens* GV2260 (pBBRacdS, pEKH_2_); virD1acdS: *A. tumefaciens* GV2260 (pvirD1acdS, pEKH_2_).

*Erianthus ravennae* calli subcultured every 2 weeks were inoculated with *A. tumefaciens* GV2260 (pBBR1MCS-5, pEKH_2_), *A. tumefaciens* GV2260 (pBBRacdS, pEKH_2_), or *A. tumefaciens* GV2260 (pvirD1acdS, pEKH_2_). The calli were cocultured in the dark for 3 days. The calli were then subcultured in callus induction medium containing 12.5 mg/L meropenem trihydrate for 1 day to eliminate *A. tumefaciens*. After the 1-day *A. tumefaciens* GV2260 elimination step, the calli were stained with GUS staining solution. Blue GUS-stained spots indicated T-DNA delivery into *E. ravennae* cells (Fig. 4A–C). To estimate the efficiency of *Agrobacterium*-mediated T-DNA delivery, the blue spots were counted. The number of blue spots per 1 g of calli was threefold higher with *A. tumefaciens* GV2260 (pBBRacdS, pEKH_2_) than with *A. tumefaciens* GV2260 (pBBR1MCS-5, pEKH_2_). ACC deaminase expression driven by the *virD1* promoter resulted in the highest number of blue spots (Fig. 4D). *A. tumefaciens* GV2260 (pvirD1acdS, pEKH_2_) resulted in 1.5-fold blue spots compared with *A. tumefaciens* GV2260 (pBBRacdS, pEKH_2_), and 4.5-fold more blue spots than the control. The effect of increasing ACC deaminase gene expression in *A. tumefaciens* on the transformation efficiency was also evaluated by measuring the GUS activity fluorometrically (Fig. 4E). Inoculation with *A. tumefaciens* GV2260 (pvirD1acdS, pEKH_2_) increased the transient GUS activity by 1.5-fold relative to that for inoculation with *A. tumefaciens* GV2260 (pBBRacdS, pEKH_2_). This result was consistent with the number of GUS-stained spots (Fig. 4D).

In this study, we increased the ACC deaminase gene expression and activity in *A. tumefaciens* GV2260 using the *virD1* promoter, which, in turn, enhanced the ability of *A. tumefaciens* to deliver T-DNA into *S. lycopersicum* and *E. ravennae* cells. By enhancing *Agrobacterium*-mediated T-DNA delivery into plant cells, the number of stable transgenic plants might be increased. Ntui et al. (2010) and Hao et al. (2010) showed that the enhancement of *Agrobacterium*-mediated T-DNA delivery into plants by the addition of ACC deaminase driven by the *lac* promoter in *A. tumefaciens* increased the stable transformation efficiency in “Egusi” melon and canola cultivars, respectively. Therefore, *A. tumefaciens* GV2260, which harbors the ACC deaminase gene driven by the *virD1* promoter and was created in this study, has the potential to increase the number of stable transgenic crop plants.

In this study, a newly produced strain of *A. tumefaciens* GV2260 (pvirD1acdS, pEKH_2_), was shown to deliver T-DNA to *E. ravennae*, which is an important biomass-producing plant, with improved efficiency. The effective production of plants for biomass that does not compete with food production is important for the utilization of biomass resources. In typical land usage patterns, the best land is used for food crop production and poor soil is used for biomass production. It is therefore necessary to make biomass-producing plants such as *Erianthus* and Sorghum tolerant to nutrient-deficient conditions through *Agrobacterium*-mediated transformation. However, reliable regeneration and transformation systems for *Erianthus* and Sorghum have not been established. *A. tumefaciens* GV2260, which harbors the ACC deaminase gene driven by the *virD1* promoter and was established in this study, might contribute to the establishment of new reliable transformation systems for biomass-producing plants.

## Supporting Information

**Table S1.** DNA primers used in this study.
