# Supplementary material for: Increased 1-aminocyclopropane-1-carboxylate deaminase activity enhances Agrobacterium tumefaciens-mediated gene delivery into plant cells
Source: Microbiologyopen. 2013 Sep 2;2(5):873–80. doi: 10.1002/mbo3.123 (PMC3831647; doi:10.1002/mbo3.123)
Supplement: Supplementary file 1 [file mbo30002-0873-SD1.doc]

Table S1: DNA primers used in this study.

| primer | Sequence (5′ to 3′) |
| --- | --- |
| acdS-*Nco*I | CCACGGTGTGCGTCCATGGG |
| acdS-*Spe*I | GCTAACTAGTTCAGCCGTCTCGGAAG |
| Plac-ATG | ATGACCATGATTACGCCAAGC |
| acdS_*Eco*RI | CGTAAGAATTCTCACTATAGGGCGAATTGG |
| GV_virB1-fusF | CGATCCATGGGGTTGCTTTGTCAGGGATGC |
| GV_virB1-fusR | CGTAATCATGGTCATACCTTATCTCCTTAGCTC |
| GV_virD1-fusF | CGATCCATGGTTTATTTCAAATTCGTTATAATTAAATTGC |
| GV_virD1-fusR | CGTAATCATGGTCATAGCTTCCTCCAAAAAAAGCG |
| GV_virE1-fusF | CGATCCATGGCGCGTGCGTCCAGTCTTTCC |
| GV_virE1-fusR | CGTAATCATGGTCATATGTTCTCTCCTGCAAAATTGC |
|  |  |
